# Radiomics analysis at PET/CT contributes to prognosis of recurrence and survival in lung cancer treated with stereotactic body radiotherapy

**DOI:** 10.1038/s41598-018-22357-y

**Published:** 2018-03-05

**Authors:** Anastasia Oikonomou, Farzad Khalvati, Pascal N. Tyrrell, Masoom A. Haider, Usman Tarique, Laura Jimenez-Juan, Michael C. Tjong, Ian Poon, Armin Eilaghi, Lisa Ehrlich, Patrick Cheung

**Affiliations:** 1 Department of Medical Imaging, Sunnybrook Health Sciences Centre, University of Toronto, Toronto, Canada; 2 Department of Medical Imaging, University of Toronto, Toronto, Canada; 3 Department of Radiation Oncology, Sunnybrook Health Sciences Centre, University of Toronto, Toronto, Canada

## Abstract

We sought to quantify contribution of radiomics and SUVmax at PET/CT to predict clinical outcome in lung cancer patients treated with stereotactic body radiotherapy (SBRT). 150 patients with 172 lung cancers, who underwent SBRT were retrospectively included. Radiomics were applied on PET/CT. Principal components (PC) for 42 CT and PET-derived features were examined to determine which ones accounted for most of variability. Survival analysis quantified ability of radiomics and SUVmax to predict outcome. PCs including homogeneity, size, maximum intensity, mean and median gray level, standard deviation, entropy, kurtosis, skewness, morphology and asymmetry were included in prediction models for regional control (RC) [PC4-HR:0.38, p = 0.02], distant control (DC) [PC4-HR:0.51, p = 0.02 and PC1-HR:1.12, p = 0.01], recurrence free probability (RFP) [PC1-HR:1.08, p = 0.04], disease specific survival (DSS) [PC2-HR:1.34, p = 0.03 and PC3-HR:0.64, p = 0.02] and overall survival (OS) [PC4-HR:0.45, p = 0.004 and PC3-HR:0.74, p = 0.02]. In combined analysis with SUVmax, PC1 lost predictive ability over SUVmax for RFP [HR:1.1, p = 0.04] and DC [HR:1.13, p = 0.002], while PC4 remained predictive of DC independent of SUVmax [HR:0.5, p = 0.02]. Radiomics remained the only predictors of OS, DSS and RC. Neither SUVmax nor radiomics predicted recurrence free survival. Radiomics on PET/CT provided complementary information for prediction of control and survival in SBRT-treated lung cancer patients.

## Introduction

Non-small cell lung cancer (NSCLC) is the leading cause of cancer-related death worldwide despite major advances in treatment^1^. Although surgical resection has been the gold standard of therapy for early stage NSCLC, newer highly focused radiation therapies have been implemented in the last decade in this population with excellent clinical outcomes that rival the results of surgical resection^2^. Stereotactic body radiotherapy (SBRT), also known as stereotactic ablative radiation therapy (SABR), has become a standard of care option for patients with early stage lung cancer who are medically inoperable or refuse to undergo surgery^2^. Although the novel technique has gained significant recognition due to its impressive high rate of local control, the dominant pattern of recurrence is that of developing distant metastases^3^. As most patients who undergo SBRT are medically inoperable due to medical co-morbidities, the overall survival rates in many published SBRT series are inferior to published surgical series due to more non-cancer deaths in those patients treated with SBRT^4,5^.

Fluorodeoxyglucose positron emission tomography/computed tomography (FDG PET/CT) is a molecular imaging technique combining metabolic and functional evaluation, which has improved the diagnostic accuracy, initial staging and restaging of lung cancer and has influenced treatment optimization and therapy response monitoring^6^. Regarding prognostication of PET/CT, most studies have shown that standardized uptake value (SUV) can be used as a prognostic indicator of survival^7–9^, while others reported that it was not an independent predictor of overall survival^10,11^.

Recently the role of radiomics in the evaluation of tumor heterogeneity has been explored based on the quantitative analysis of medical image data^12–16^. The rationale behind this concept is that advanced image analysis may capture additional information regarding the prognosis of a tumor based on a pre-treatment imaging study that would guide decision-making towards the most beneficial personalized treatment. The combination of radiomics and more classical PET parameters has been studied in the SBRT treated lung cancer population in a few studies but these studies have either focused solely on distant metastasis as a clinical outcome^17^ or failed to predict overall survival^18^. Furthermore, no study to date has evaluated radiomics in both the CT component and PET component of integrated PET/CT images in patients with lung cancer treated with SBRT.

We hypothesized that radiomic features on both the CT and PET components of PET/CT were associated with clinical outcomes and survival and that adding radiomic features to the classic imaging parameters of PET (e.g., SUVmax) would improve prognostication of N0M0 lung cancer patients post SBRT.

## Results

At two years, the Kaplan-Meier estimate of the percentage of local control (LC) was 95%, lobar control (LOBC) was 92%, regional control (RC) was 90%, distant control (DC) was 75% and recurrence free progression (RFP) was 69%. Two-year recurrence free survival (RFS) was 69%, overall survival (OS) was 79% and disease specific survival (DSS) was 88% (Table 1).

**Table 1:** Characteristics of the patients, lesions and treatment.

Patients	150
Male/Female	73/77
Age	74 (46–92)
T-stage	
T1aN0	62
T1bN0	51
T2aN0	33
T2bN0	2
T3 N0	25
Histology	130
Adenocarcinoma	69 (including 5 former BAC)
Squamous	37
large cell	1
NSCLC undifferentiated	11
non-diagnostic biopsy	12
Not biopsy proven – (judged NSCLC in multidisciplinary tumour board meetings)	42
Size (cm)	2.4 (0.7–5.8)
Location	
Upper	102 (59.3%)
Middle	11 (6.39%)
Lower	59 (34.3%)
Patients w 1 lesion	130
Patients w 2 lesions	18
Patients w 3 lesions	2
Follow-up period (mo)	Mean: 28 (3–66), Median: 27
Total dose – Gy (nr pts)	
48	107/172
52	51/172
50	13/172
56	1/172
Outcomes at 2 years	
local control (LC)	95% (CI: 91–99%)
lobar control (LOBC)	92% (CI: 87–97%)
regional control (RC)	90% (CI: 85–95%)
distant control (DC)	75% (CI: 67–83%)
recurrence free probability (RFP)	69% (CI: 62–77%)
recurrence free survival (RFS)	69% (CI: 61–77%)
overall survival (OS)	79% (CI: 72–86%)
disease specific survival (DSS)	88% (CI: 82–93%)

As this is an exploratory and hypothesis generating study we have included principal components which might have lower clinical relevance (eigenvalue <1) but could prove to contribute significantly to our predictive model. In this context, based on CT and PET images of PET/CT the top radiomic features that were most heavily weighted in the principal components and were found to be significant predictors in the clinical outcome/survival analysis are described below. Overall we found 4 PCs (PC1-PC4)-each one including 6–8 different radiomic features-to be significant and enter predictive models of OS, RFP, DC, DSS and RC.

**Figure 1:**
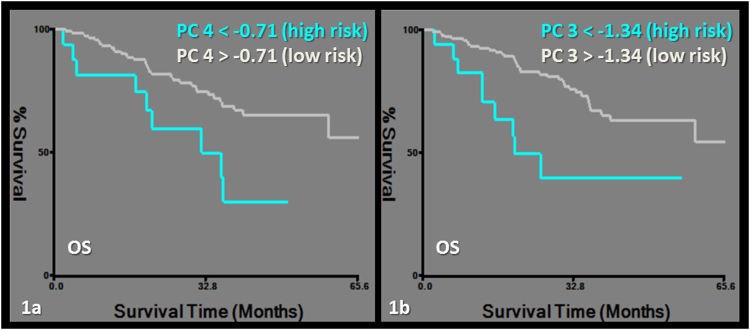
Kaplan-Meier survival curves for overall survival (OS). Subgroups of low and high risk were determined by a cut-off value of −0.71 for PC4 (logrank chi-square: 7.39, p = 0.09) (**a**) and −1.34 for PC3 (logrank chi-square: 8.92, p = 0.002) (**b**).

OS was predicted by a model including PC4 and PC3. Exclusively PET-derived features including first order (kurtosis and skewness), second order features (homogeneity and normalized entropy) and morphological features (morphology1 and 2) were grouped in PC4. Morphology1 assesses the area irregularity and morphology2 assesses the perimeter irregularity^19^. PC3 included exclusively CT-derived first order and morphological features (morphology2, asymmetry1 and 3) (Tables 2, 3, 4, Fig. 1). The asymmetry feature group measures the degree of bilateral symmetry exhibited by the lesion^19^.

**Table 2:** Clinical outcome/Survival analysis with Principal components and without or with SUVmax.

	OS	RFP	DC	RC	DSS
Without SUVmax (HR [CI]), p value	PC 4 *0.45 [0.26–0.78]*, *p* = *0.004*	SEX *2[[1.11–3.62]*, *p* = *0.02*	PC 4 *0.51 [0.28–0.91]*, *p* = *0.02*	PC 4 *0.38 [0.17–0.83]*, *p* = *0.02*	PC 2 *1.34 [0.09–17.88]* *p* = *0.03*
	PC 3 *0.74 [0.58–0.96]*, *p* = *0.02*	PC 1 *1.08 [1.00–1.17]*, *p* = *0.04*	PC 1 *1.12 [1.02–1.23]*, *p* = *0.01*		PC 3 *0.64 [0.43–0.94]* *p* = *0.02*
With SUVmax (HR [CI]), p value	PC 4 *0.45 [0.26–0.78]*, *p* = *0.004*	SEX *2.06 [1.14–3.73]*, *p* = *0.02*	PC 4 *0.5 [0.28–0.91]*, *p* = *0.02*	PC 4 *0.38 [0.17–0.83]*, *p* = *0.02*	PC 2 *1.34 [0.09–17.88]* *p* = *0.03*
	PC 3 *0.74 [0.58–0.96]*, *p* = *0.02*	SUV *1.1 [1.03–1.18]*, *p* = *0.04*	SUV *1.13 [1.04–1.22]*, *p* = *0.002*		PC 3 *0.64 [0.43–0.94]* *p* = *0.02*

OS: Overall survival, RFP: Recurrence free probability, DC: Distant control, RC: Regional control, DSS: Disease specific survival.

**Table 3:** Summary of texture feature groups.

Feature group	Number of features	Description
Statistical-First order^16^	8	Region of interest Size_# of pixels (ROI Size), Mean gray level, Standard Deviation (SD), Median gray level, Region of interest_Minimum pixel intensity (ROI Min), Region of interest_Maximum pixel intensity (ROI Max), Kurtosis, Skewness
Textural-Second order^15,16,28^	6	Contrast, Energy, Correlation, Homogeneity, Entropy, Normalized Entropy
Morphology^19^	3	Area regularity (1), perimeter regularity (2)
Asymmetry^19^	4	Region bilateral symmetry (4)

**Table 4:** Significant Principal Components of Textural features based on PET/CT.

PC Features	PC1 (eigenvalue 15.17)	PC2 (eigenvalue 2.79)	PC3 (eigenvalue 1.37)	PC4 (eigenvalue 0.31)
Statistical-First order	ROI max pixel intensity (*PET*)	ROI max pixel intensity (CT)	ROI max pixel intensity (CT)	Kurtosis (*PET*)
	ROI size (*PET*)	Standard Deviation (CT)	Mean Gray Level (CT)	Skewness* (*PET*)
	Mean Gray Level (*PET*)	Mean Gray Level (CT)	Median Gray Level (CT)	
	Median Gray Level (*PET*)		Standard Deviation (CT)	
	Standard Deviation (*PET*)			
Textural-Second order	Homogeneity (CT)			Homogeneity (*PET*)
	Normalized Entropy* (CT)			Normalized Entropy (*PET*)
	Entropy* (CT)			
Morphologic		Asymmetry1 (CT)	Morphology2 (CT)	Morphology1 (*PET*)
		Asymmetry2 (CT)	Asymmetry1* (CT)	Morphology1 (CT)
		Asymmetry3 (CT)	Asymmetry3* (CT)	Morphology2* (*PET*)
		Asymmetry4 (CT)		

“*”Indicates the negative correlation of the specific feature.

**Figure 2:**
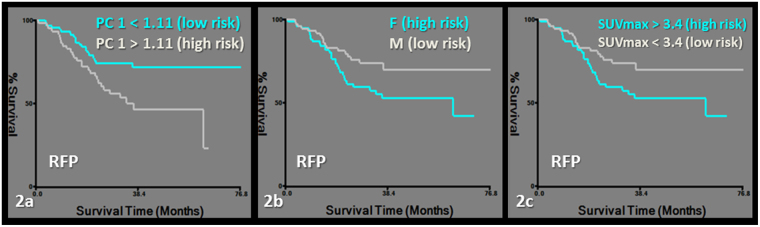
Kaplan-Meier survival curves for recurrence free probability (RFP). Subgroups of low and high risk were determined by a cut-off value of 1.11 for PC1 (logrank chi-square: 7.09, p = 0.007) (**a**), female gender (logrank chi-square: 3.82, p = 0.05) (**b**) and a cut-off value of 3.4 for SUVmax (logrank chi-square: 6.75, p = 0.009) (**c**).

**Figure 3:**
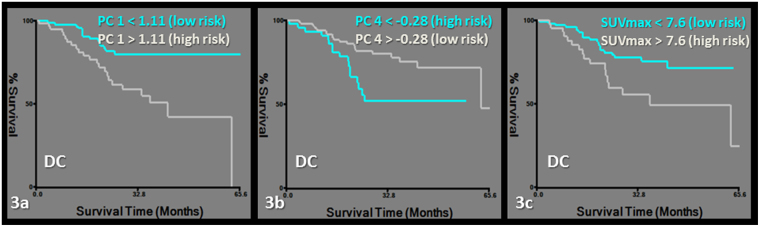
Kaplan-Meier survival curves for distant control (DC). Subgroups of low and high risk were determined by a cut-off value of 1.11 for PC1 (logrank chi-square: 11.62, p = 0.0006) (**a**), −0.28 for PC4 (logrank chi-square: 6.27, p = 0.01) (**b**) and 7.6 for SUVmax (logrank chi-square: 6.22, p = 0.01).

RFP was predicted by a model including PC1 and female predominance in gender. Features grouped together in PC1 were PET-derived first order features and CT-derived second order features (Tables 2, 3, 4, Fig. 2). DC was predicted by a model including PC4 and PC1 (Tables 2, 3, 4, Fig. 3).

**Figure 4:**
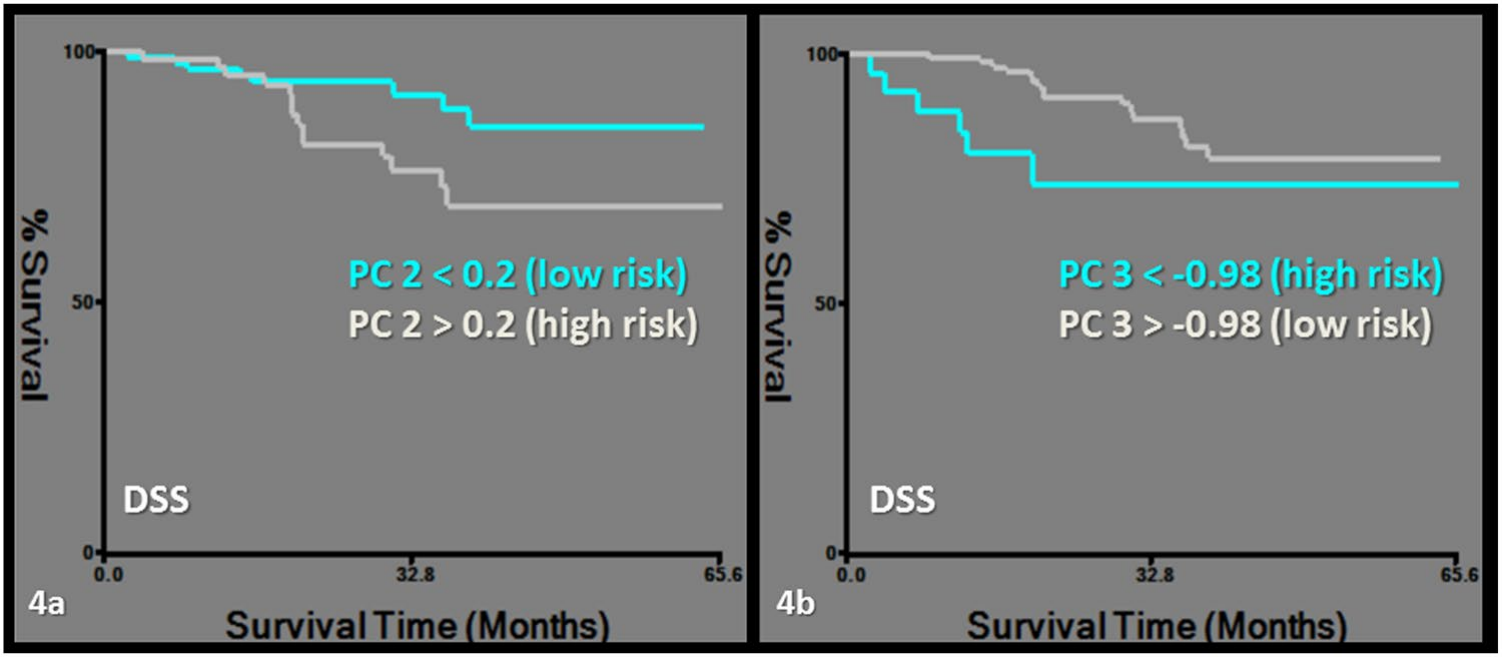
Kaplan-Meier survival curves for disease specific survival (DSS). Subgroups of low and high risk were determined by a cut-off value of 0.2 for PC2 (logrank chi-square: 4.08, p = 0.04) (**a**) and −0.98 for PC3 (logrank chi-square: 4.21, p = 0.04) (**b**).

**Figure 5:**
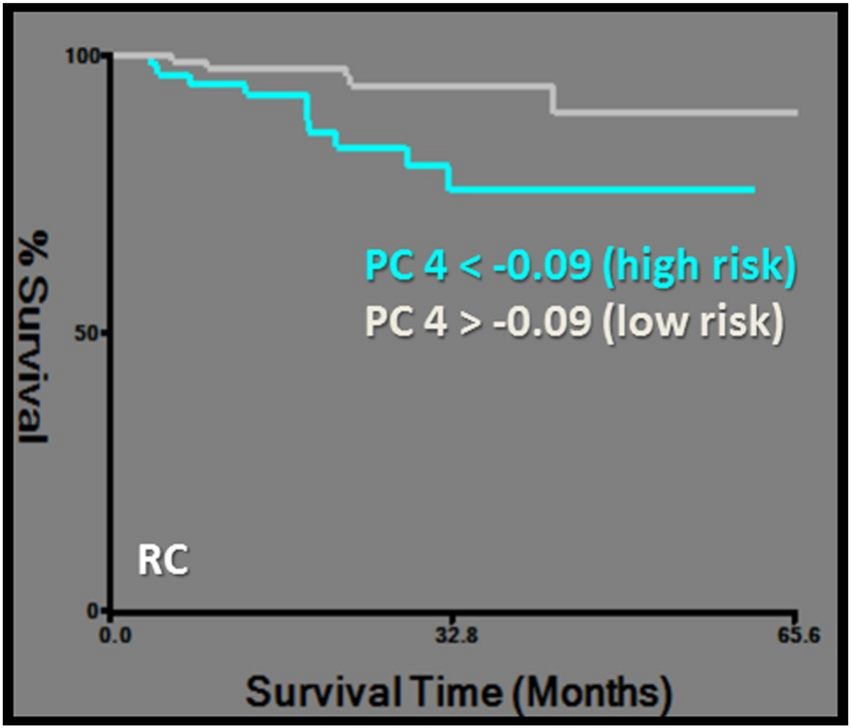
Kaplan-Meier survival curve for regional control (RC). Subgroups of low and high risk were determined by a cut-off value of −0.09 for PC4 (logrank chi-square: 6.19, p = 0.01).

DSS was predicted by PC2, which was exclusively based on CT-derived first order and morphological features, namely the asymmetry group. The same first order features were also clustered in PC1 and PC2 (Tables 2, 3, 4, Fig. 4). RC was predicted by PC4, which is described above (Tables 2, 3, 4, Fig. 5).

Finally, the added value of radiomics analysis in staging PET/CT was tested by rerunning the clinical outcome/survival analysis after entering SUVmax with the principal components, age, female predominance in gender, histology, stage and radiation dose. PC1 lost its predictive value over SUVmax for the models of RFP and DC. Female gender remained predictive of RFP and PC4 remained predictive of DC independent of SUVmax in the models (Table 2, Figs 2 and 3).

SUVmax was not predictive of OS, DSS and RC. No principal component, neither the SUVmax were significant predictors of RFS in the clinical outcome/survival analysis. None of the remaining clinical parameters (age, histology, stage, radiation dose) remained in the predictive models when they were entered into a multivariable regression analysis.

## Discussion

In this study, we identified CT and PET-derived principal components of radiomic features reflecting heterogeneity and morphology based on staging PET/CT of patients with lung cancer candidates for SBRT, which were predictors of RC, DC, RFP, DSS and OS. In a combined analysis including principal components and SUVmax, the latter was included in the models and was predictive only of DC and RFP.

To our knowledge this study is the first one to explore the predictive ability of radiomics based on both the CT and PET component of staging PET/CT in early stage lung cancer patients and to report on the complementary role of radiomics and SUVmax in predicting clinical outcome and overall survival. Two other recent studies have addressed the role of radiomics in SBRT treated early-stage lung cancer population, however these studies had a smaller sample size and were solely based on PET images^17,18^ or explored the prediction only of DC^17^. A third recent study explored the role of diagnostic CT-derived radiomics signature of early stage (I and II) lung cancer in predicting disease free survival^20^.

Evaluating a homogeneous population with N0M0 lung cancer treated with a specific radiation therapy such as SBRT is advantageous compared to other studies which assessed a heterogeneous group of patients with different stages of lung cancer, as it adds complementary predictive information beyond the stage of the patient. Patients treated with SBRT are unique in that they sometimes lack specific pathologic confirmation of the tumor itself, and most do not undergo invasive mediastinal staging. Microscopic pathologic involvement of the regional nodes (which may not be detected on any current imaging modality)-is a known adverse prognostic factor. Therefore, it is of paramount importance to identify other surrogates in this N0M0 lung cancer population that could predict recurrence and survival in order to select patients who would benefit from more aggressive additional treatment other than SBRT, such as surgery or adjuvant chemotherapy.

Due to the large number of extracted radiomics features from the CT and PET component of PET/CT studies and due to high correlation between each other, we chose to perform unsupervised feature reduction using the principal components analysis (PCA). PCA selects a small number of uncorrelated variables that maintain the interaction amongst them when they are combined and that could explain most of the variation in the data^21^.

We identified specific principal components based on CT and PET-derived radiomics features that were included in prediction models of RC, DC, RFP, DSS and OS. Recurrence free probability (RFP) was predicted by a model including PC1 and female gender. PC1 included PET-derived first order features and CT-derived second order features, namely homogeneity, normalized entropy and entropy. First order features describe the distribution of values of individual voxels without taking into account spatial relationships and are based on histogram analysis measuring intensity of the image^22^. The size of the tumor included in PC1 has been found to be a significant predictor of survival in lung cancer^23^ and in our study tumor size was included in the predictive model of RFP and DC. Homogeneity is the opposite of contrast in a given window^16^. In another study, CT-derived homogeneity together with kurtosis and uniformity were included in the radiomics signature predicting disease free survival, for which we did not identify any predictors in our study^20^. Entropy reflects the randomness of gray-level voxel intensities within an image^16^. Normalized entropy is the ratio of entropy to size (of ROI) to account for the degree of heterogeneity with respect to the size of the tumor and has been found to be a predictor of survival in NSCLC patients^24^.

Distant control (DC) was predicted by a model including PC1 and PC4. Radiomics features grouped in PC4 were all PET-derived including first order kurtosis and skewness, second order homogeneity and normalized entropy and morphological features. Kurtosis measures the flatness of the histogram of values^22^, while skewness measures the asymmetry of the histogram^16^. Lower kurtosis and positive skewness were found to be significantly associated with k-ras mutations in one study^25^. In the same study kurtosis was prognostic for overall survival and disease free survival^25^. In our study lower kurtosis and positive skewness are associated with higher distant metastatic disease. Morphology1 and morphology2 features capture the characteristics of the ROI shape where the former manipulates the shape (area) and the latter manipulates the perimeter to calculate the regularity/irregularity of the ROI shape^19^.

Overall survival (OS) was predicted by a model including PC4 and PC3. Percentage change in PET-derived entropy, which is clustered in PC4 in our study, was an independent predictor of overall survival in patients with lung adenocarcinoma^15^. In another study PET-derived entropy was an independent predictor of disease specific survival^18^ and PET-derived correlation was an independent predictor of OS^18^. PC3 which was predictive of OS included CT-derived first order features that were also seen in PC1 and PC2 and CT-derived morphological features including morphology and asymmetry. The asymmetry feature group measures the degree of bilateral symmetry exhibited by the lesion. Four different asymmetry features are calculated based on the normalized difference in shape of the 2 components of the lesion by splitting it along the minor or major axis and by choosing either the entire region area or the area of the smaller half region to normalize. Although these features have been used for detection of prostate cancer, they have not been previously assessed in radiomics analysis of lung cancer^19^.

Disease specific survival (DSS) is an important clinical outcome for SBRT treated patients since this population is largely affected by other comorbidities which may occasionally be the cause of death and not lung cancer^4,5^. DSS was predicted by PC2 that included exclusively CT-derived features. These were first order features, which were also clustered in PC1 and PC3, or morphological features, namely asymmetry1–4. PC2 was very closely associated with PC3 as they both share common CT-derived first order features and morphological features. Pyka *et al*. found that only entropy was predictive of DSS and Lovinfosse *et al*. reported that only dissimilarity was predictive of DSS^18,26^.

CT texture features of heterogeneity in lung cancer have been reported to correlate with markers of hypoxia and angiogenesis^27^. Our study was in agreement with that and showed that CT and PET-derived homogeneity (grouped in PC1 and PC4) were predictive of DC and OS. Ganeshan *et al*.^28^ found that CT-derived uniformity and PET-stage were the only independent predictors of OS in lung cancer. Differences in the significance of specific textural features may partly be attributed to the fact that those studies used diagnostic CTs to extract radiomic features, while we used the low dose CT component of the PET/CT studies in order to make the most out of the combined information given from the integrated PET/CT studies. The only other study that attempted to do the same was the one by Win *et al*.^24^, which reported that heterogeneity derived from the CT component of PET/CT and CT-derived permeability together with the clinical stage were the only predictors of OS. No PET-derived radiomics features were found to predict for clinical outcome. However, in our study principal components of both CT and PET-derived radiomics features were included in prediction models of clinical outcome.

Our study also showed that SUVmax was included in the prediction models of DC and RFP when combined with PCs based on radiomic features. Chang *et al*.^29^ reported that SUVmax was a multivariate predictor of OS in patients with early stage lung cancer treated with SBRT. SUVmax was a univariate predictor of DC and a multivariate predictor of RFS^24^. Consistently, Takeda *et al*. reported that pre-SBRT SUVmax was a predictor of local control^30^ and Satoh *et al*. reported that it was a predictor of disease free survival^31^. On the other hand, other authors reported that SUVmax did not predict for any clinical outcome in SBRT population^18,32,33^. In studies comparing texture analysis and classical PET parameters Ganeshan *et al*.^28^ found that SUVmax did not predict OS in lung cancer patients, Satoh *et al*.^33^ reported no correlation of SUVmax with any clinical outcome and Pyka *et al*.^18^ and Lovinfosse *et al*.^26^ found no correlation of the examined PET parameters with clinical outcomes in lung cancer patients treated with SBRT.

It is noteworthy that when SUVmax was combined with the PCs in the analysis, PC1 lost its predictive value over SUVmax for DC and RFP. PC1 includes PET-derived first order features, apart from CT-derived homogeneity, entropy and normalized entropy. SUVmax and first order features both represent “intensity characteristics of the voxels” irrelevant of their relationship with the neighboring voxels. Therefore, in the combined analysis, SUVmax appears to be stronger predictor compared to the first order features (represented by PC1). However, in the combined analysis, PC4 remained predictive of DC independent of SUVmax, probably reflecting that PET-derived second order and morphological features grouped in PC4 represent different characteristics of the tumor compared to SUVmax. Moreover, SUVmax was not predictive of OS, DSS and RC.

The female gender in our study was found to have an unfavorable predictive outcome regarding RFP. Although female gender has been associated with longer overall survival and good prognosis in SBRT for NSCLC^26^, some authors have reported that 4 out of 5 patients with SBRT treated lung cancer who presented with late recurrence were women^34^. In our study the median follow up period was 27 months with maximum period of follow up being 66 months (>5 years) which may have accounted for detection of higher rate of late recurrence in female patients. Other studies have not identified gender as significant predictor in SBRT lung cancer patients^35^. Unlike Huang’s *et al*. study, in our study none of the other clinical parameters including age, histology, stage and radiation dose (BED) were found to be significant predictors of any clinical outcome studied^20^. Differences may partly be explained by Huang’s study was based on a longer period for estimation of survival outcome (3 years versus 2 years in our study)^20^.

Our study has several limitations. The study was retrospective and a few of the patients did not have histologic confirmation of the primary tumor as is the case in many studies based on SBRT treated lung cancer patients. Some SBRT-treated patients did not undergo PET/CT for staging purposes and therefore could not be included in the study. A further limitation was that we included 20 patients with more than one lesion treated with SBRT in the overall population. However, the clinical outcome evaluated per patient was based on the CT and PET-derived radiomics features of the dominant lesion, which was the one with the highest SUVmax. Another limitation may be related to the free breathing as opposed to respiratory gating used for the acquisition of PET images, which may have influenced the quantification of lung lesions. However comparison of free breathing and respiratory gated PET images did not show any significant differences in textural parameters of lung tumors^36^. As this is an exploratory and hypothesis generating study we chose to include principal components which might have lower clinical relevance (eigenvalue <1) but could contribute significantly in our predictive model. Finally, inter-observer reliability was not tested in the current study but has been found to be robust in other studies related to radiomics analysis based on imaging of lung cancer^15,21^.

Future goals would include validation of these results in larger prospective cohorts of patients and longer follow-up times and application in other homogeneous lung cancer populations, such as early stage lung cancer treated with surgery as opposed to SBRT.

In conclusion, we have identified prediction models of RC, DC, RFP, DSS and OS based on radiomic features derived from the CT and PET component of staging PET/CT in early stage lung cancer patients treated with SBRT. The combination of a classic PET/CT parameter-SUVmax-and radiomic signatures resulted in prediction models of DC and RFP. SUVmax failed to predict OS, DSS and RC and neither SUVmax nor radiomic features predicted RFS. Adding radiomic features in staging PET/CT improves the prognostication in early stage lung cancer patients treated with SBRT and may impact decision-making for identifying patients who will benefit from adjuvant therapy or even surgery.

## Methods

This study was approved by the Research Ethics Board of Sunnybrook (REB) Health Sciences Centre (project ID: 077-2014) and all methods were carried out in accordance with relevant guidelines and regulations. The study was based on a retrospective lung SBRT database of a cohort of 267 patients treated between April 2008 and September 2012 from our Institution^35^. All SBRT procedures and PET/CT studies were performed at one Academic Hospital (Sunnybrook Health Sciences Centre, Toronto, Canada) and this was a single institution retrospective study. The Sunnybrook REB determined that an informed consent form was not required for this study as it was retrospective, and therefore informed consent was not obtained as it was waived by REB.

### Patients

Out of the 267 patients treated with SBRT at our institution between April 2008 and September 2012, 212 had primary N0M0 lung cancer and the remaining 55 had pulmonary metastases. 62 out of the 212 patients with primary lung cancer were excluded because they did not have pre SBRT PET/CT imaging. 150 patients (77 female and 73 male) with mean age 74 years (46–92) and overall 172 lesions, with stage T1-T3 N0M0 lung cancer were included in the study (Table 1). They all had undergone pre-treatment staging PET/CT imaging as part of a work-up for their primary lung tumors and were subsequently treated with SBRT. All patients were medically inoperable due to other comorbidities (n = 127) or refused surgery (n = 23) and underwent SBRT treatment. Clinical data about tumor histology, primary tumor size, prescribed biological effective dose, age, gender, initial stage, local recurrence, regional recurrence, distant recurrence and death were obtained from the institutional database and are summarized in Table 1.

Ideally, patients had pathologic confirmation of lung cancer. If pathologic confirmation was not possible, then there had to be significant (FDG) activity on positron emission tomography – computed tomography (PET/CT) defined by a maximum standardized uptake value (SUVmax) ≥2.5 or evidence of tumor growth in at least two serial CT scans. Patients with a past diagnosis of lung cancer were categorized as having a second primary NSCLC if there was confirmation of a different NSCLC subtype, or if a new malignant nodule developed more than two years after the initial diagnosis of lung cancer with no evidence of distant metastatic disease. All other new FDG-avid or enlarging solid lung nodules occurring within the first 2 years after the initial diagnosis of lung cancer were considered lung metastases from NSCLC. 118 tumors were biopsy proven based on transbronchial or CT-guided biopsy. 12 tumors underwent inconclusive biopsy and the remaining 42 tumors did not undergo biopsy and were judged to be non-small cell lung cancer by consensus in multidisciplinary tumor board meetings based on serial CT and PET-CT findings. The median follow-up period after SBRT was 27 months (3–66).

The FDG-PET/CT technique and SBRT method can be found as Supplementary File.

### Texture analysis on CT and PET images of PET/CT studies

**Figure 6:**
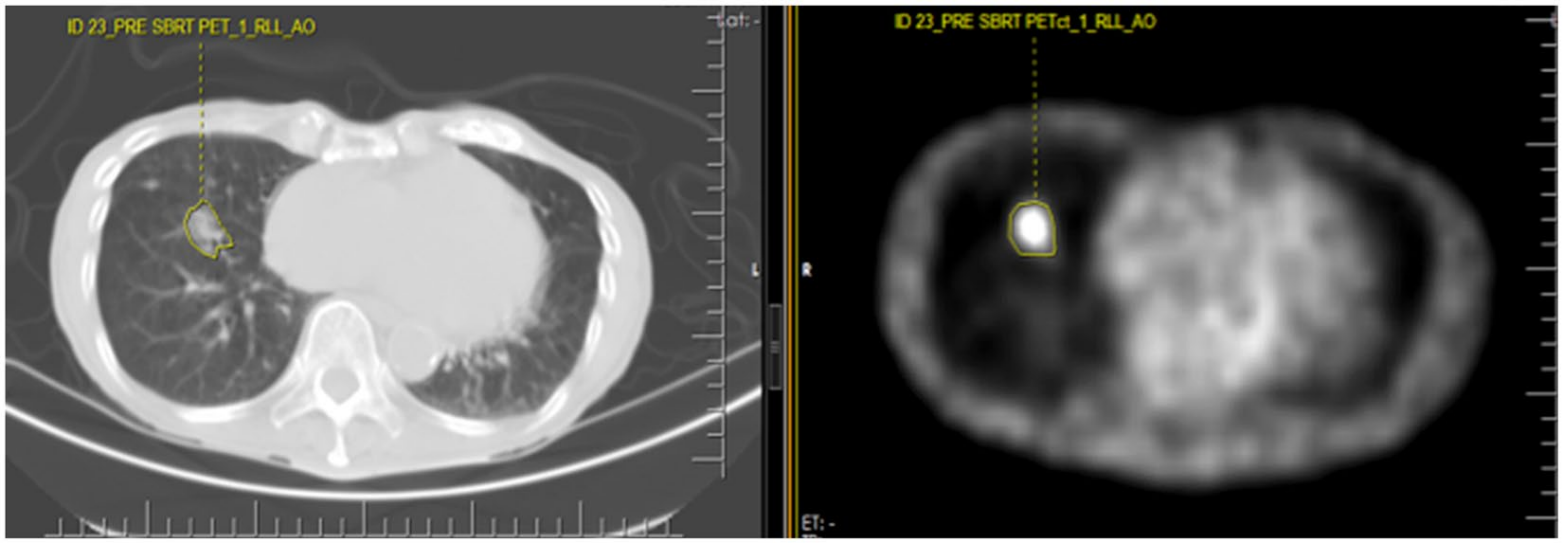
Screenshot of the texture analysis software applied on a staging PET/CT study for a NSCLC patient before SBRT therapy. On the left is the CT image and on the right, is the PET image of the PET/CT at the exact same level. The manual contouring of the right lower lobe tumor on both images is noted. There was an event of distant metastasis and death. SUVmax = 1.9. The significantly low SUVmax failed to predict the poor clinical outcome as evidenced by the development of distant metastasis and ultimately death.

**Figure 7:**
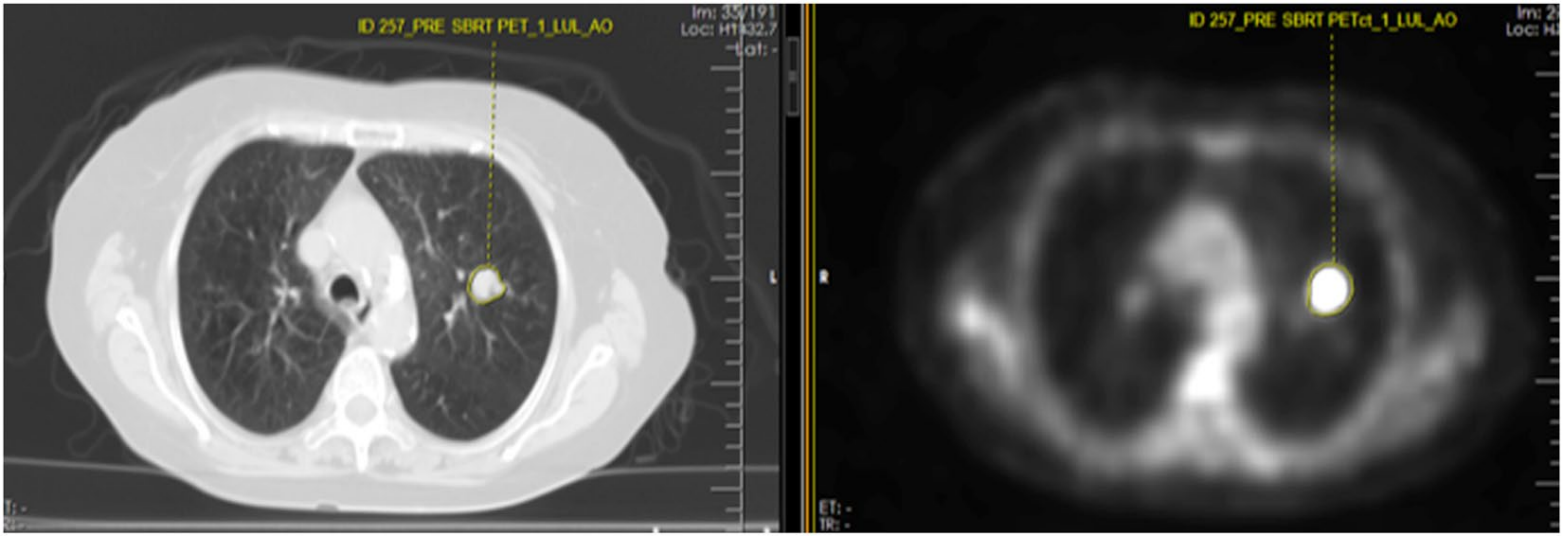
Screenshot of the texture analysis software applied on a staging PET/CT study for a NSCLC patient before SBRT therapy. On the left is the CT image and on the right, is the PET image of the PET/CT at the exact same level. The manual contouring of the tumor on both images is noted. There was no clinical event. SUVmax = 11.4. In comparison to the patient in the Fig. 1, the higher SUVmax did not correlate with the absence of clinical event.

The CT and CT corrected attenuated PET images were transferred to a dedicated research computer for further textural feature analysis with ProCanVAS (Prostate Cancer Visualization and Analysis System), a computer aided imaging diagnosis tool developed in our department. Preliminary image thresholding was performed to exclude air, fat tissue and calcifications, i.e. pixels with attenuation values <−50 HU and >300 HU were excluded. Manual contouring was performed on the lesion of interest separately on the CT and PET images of the pre-treatment PET/CT studies. The delineation of the tumors was performed manually by a thoracic radiologist (A.O.) with 14 years of experience in thoracic imaging and 2 years of experience in texture analysis of lung cancer with the help of a 3rd year medical student (U.T.) involved in the research project. Each lesion was contoured on every sequential slice that was visible on CT as increased homogeneous or ground glass density compared to surrounding normal lung parenchyma. Attention was made so that volume averaging areas, adjacent vascular structures were not included in the regions of interest. The segmentation/contouring of the lesions on the PET images was performed manually on all the sequential images showing increased FDG uptake in the corresponding area of the tumor, which was either the same area covered on the equivalent CT images or slightly smaller. The texture features were calculated for each separate slice (Figs 6 and 7) on the PET and CT images.

Twenty-one texture parameters were calculated (Table 3). 8 bins were used when calculating the second-order features and relative resampling was done with respect to the ROI at hand. The median values of texture features were used for statistical analysis, which were the median of the values of texture features calculated from all the slices where the tumor was visible. The features were calculated on 2D ROIs of individual slices. The detailed list of textural features is summarized in Table 3.

For PET images, a correction factor was applied in order to convert the PET image counts per voxel to SUV units according to the activity concentration in the tissue, to the administered activity and bodyweight for each patient. Reproducibility for PET-derived textural features in lung cancer has been reported to be similar or better than that for SUVs^15,37^.

### Evaluation of patient outcomes

Patients were followed-up with CT of the chest and abdomen every 4 months for the first 3 years after SBRT and every 6 months thereafter. Local and lobar control was assessed for each pulmonary lesion treated. Regional, distant control and overall survival were calculated based on each patient treated. For the 20 patients that had more than one lesion treated with SBRT, only the texture features and SUVmax of the dominant lesion used in the statistical analysis. The dominant lesion was considered the one with the highest SUVmax value. Time to recurrence and overall survival were calculated from the start of the SBRT to the date of the event or final follow-up visit. Local control (LC) was defined as absence of relapse within the area of the planning target volume (PTV), under the condition that there was no evidence of consecutive enlargement of the lesion over 2–3 CT scans or if there was absence of tissue biopsy that confirmed a positive result. Lobar control (LOBC) was defined as absence of relapse within the same lobe of the irradiated tumor. Regional control (RC) was defined as absence of recurrence in hilar or mediastinal lymph nodes. Distant control (DC) was defined as absence of recurrence outside of local, lobar or regional recurrences^19,38^. Recurrence free probability (RFP) was defined as the absence of any recurrence. Recurrence free survival (RFS) was defined as the time from SBRT treatment to the earliest of recurrence (local, lobar, regional, distant), second cancer, death or final follow-up visit^39^. Disease specific survival (DSS) was defined as the time from SBRT treatment to the time of lung cancer-related death^26^.

### Statistical analysis

For each type of control and for recurrence-free survival and overall survival, the proportion of patients remaining event-free during the follow-up period was estimated using the Kaplan-Meier method.

We examined the principal components defining the CT and PET component of staging PET/CT for evaluation of lung cancer for the 42 features extracted to determine which features accounted for most of the variability (PCA node on the SAS Enterprise Miner system). Relationships between features were assumed to be linear, or at least approximately so, and principal components analysis (PCA) rotated the original data to new coordinates, making the data as “flat” as possible. Each principal component was defined as a linear combination of the 42 original features and could be interpreted based on the weights associated with each of these features. All feature data was centered and scaled before entering into PCA. We restricted our subsequent analysis to the first 18 principal components (96% of total eigenvalue) based on interpretation of the scree plot and incorporated these new features to the study cohort data set for further analyses (Table 4). Clinical outcomes of LC, LOBC, RC, DC, RFP, RFS, DSS and OS were modeled using Kaplan-Meier analysis with Cox proportional regression analysis to determine the significance of predictors (Survival node on the SAS Enterprise Miner system). Findings from this analysis are reported as hazard ratio (HR) with 95% confidence interval (CI). From the univariable regression models described, multivariable models were created. Those 18 PCA components as well clinical parameters including age, gender, histology, stage and radiation dose (biological equivalent dose-BED) were entered into a multivariable regression analysis with stepwise selection of variables to obtain a final model. This was also repeated for all models with the inclusion of SUVmax separately. Underlying assumptions of all Cox proportional hazard regression models were checked and satisfactory. We performed an internal validation using the 10-fold cross validation method in order to assess the stability of our primary PCA results. Overlap was observed between the principal components of the 10 repetitions with any minor discrepancies in overlap not resulting in a change of interpretation. This suggested that our primary PCA results were acceptably stable.

The patients were stratified into high-risk or low-risk groups according to the Rad-score, the threshold of which was calculated by using X-tile^40^. Differences in the survival curves of the high-risk and low-risk groups were then compared using Kaplan-Meier curves and the log-rank test.

A two-sided level of significance with a p value of less than 0.05 was used for all tests and because the analyses are exploratory, no adjustment was made for multiple comparisons. All statistical analyses described above were performed using SAS Enterprise Miner version 14.1 for windows and SAS version 9.4 for windows (SAS Institute, Cary, North Carolina, USA).

### Data availability statement

The datasets generated during and/or analyzed during the current study are available from the corresponding author on reasonable request.

## Electronic supplementary material

Supplementary information
