# Supplementary material for: Radiomics analysis at PET/CT contributes to prognosis of recurrence and survival in lung cancer treated with stereotactic body radiotherapy
Source: Sci Rep. 2018 Mar 5;8:4003. doi: 10.1038/s41598-018-22357-y (PMC5838232; doi:10.1038/s41598-018-22357-y)
Supplement: Supplementary file 1 — Supplementary information [file 41598_2018_22357_MOESM1_ESM.docx]

**Manuscript Title:**

Radiomics analysis at PET/CT contributes to prognosis of recurrence and survival in lung cancer treated with stereotactic body radiotherapy

1. *Anastasia Oikonomou, MD, PhD
2. Farzad Khalvati, PhD
3. Pascal N Tyrrell, PhD
4. Masoom A Haider, MD, FRCPC
5. Usman Tarique, BHSc, MD
6. Laura Jimenez-Juan, MD
7. Michael C Tjong, MD
8. Ian Poon, MD, FRCPC
9. Armin Eilaghi, PhD
10. Lisa Ehrlich, MD, FRCPC
11. Patrick Cheung, MD, FRCPC

**FDG-PET/CT studies**

PET/CT was performed in all patients within 6 weeks before SBRT. All images had been acquired using a PET/CT scanner (Philips GEMINI PET/CT, Philips Health Care, Cleveland, Ohio, USA) with both CT and PET data acquired in one procedure in accordance with a standardized protocol. The use of integrated PET-CT ensured precise spatial correlation between the PET and CT images.

All patients had fasted for 6 hours before the PET/CT examination and received an intravenous injection of 5.5 MBq/kg FDG of body weight to a maximum of 370 MBq/patient when it was confirmed that serum glucose was confirmed to be less than 200 mg/dL or less than 260 mg/dL if they had fasted for more than 6 hours and have not received insulin during the last 2 hours prior to FDG injection. PET-CT images were acquired in the supine position from the center of the skull up to the upper thigh, 60 minutes (range 50 - 70) after the FDG injection. CT images were acquired for attenuation correction including a conventional unenhanced CT from the base of the skull to the upper thighs (100 mAs, 120 kVp, rotational speed=1 s/rot, pitch=1, slice thickness=3.0 mm, field of view: 60 cm for attenuation correction followed by a second reconstruction of the same data for visualization at 45 cm). The patients were free breathing during the procedure. The PET images were acquired immediately after the CT studies, with a 57.6 cm transverse field of view in a 3-dimensional mode. Transaxial matrix sizes were 512x512 and 144x144 for CT and PET, respectively. After the transmission scan, the PET acquisition time was 3 minutes for each table position. The PET values were normalized using the patient’s weight divided by the decay-corrected administered activity to derive SUVs [SUV = Activity concentration in the tissue [Bq/g] / {Administered activity [Bq] / body weight [g]}]. The matrix of the CT data was then changed from 512 x 512 to 144 x 144, to match the PET data so that images could be fused and CT-based transmission maps could be generated. PET data were reconstructed iteratively using the 3D-row action maximum likelihood algorithm (3D-RAMLA) implemented by the manufacturer including Monte Carlo single-scatter simulation, decay correction and attenuation correction based on the CT data. Integrated, co-registered PET/CT images in axial plane were reviewed at a workstation (GEMINI workstation with Syntegra Image Fusion, Philips Medical Systems, Cleveland, Ohio) that enabled image fusion and analysis. A nuclear medicine physician (L.E.) with 12 years of experience in reading PET/CT retrospectively evaluated the FDG uptake of the tumors and maximum standardized uptake value (SUVmax) based on region-of interest (ROI) 3D analysis by using attenuation-corrected images. A circular ROI was initially placed by the user on a most representative transaxial FDG-PET image including the tumor using tumor-PET Viewing software, Philips Extended Brilliance Workspace. Then its diameter was manually increased to ensure that the entire tumor area was included and that the corresponding 3D volume would encompass the highest value voxel in the tumor, considering voxels in all 3 directions. Care was taken that this VOI did not cover any areas of high uptake outside of the tumor. The maximum voxel value within the spherical volume was used to derive the SUV max [1].

**SBRT Method**

The SBRT technique at our institution has been previously described [2]. Patients were immobilized using one of 2 techniques: the Elekta BlueBAG vacuum cushion (Elekta AB, Stockholm, Sweden) with an abdominal compression plate, or the full Elekta BodyFIX system. A four dimensional (4D)-CT was acquired with phase-binning reconstruction software. The gross tumor volume (GTV) was delineated by the radiation oncologist on the 0% (peak inspiratory), 50% (peak expiratory), and maximum intensity projection (MIP) image sets, and their combined volume was used to generate the internal target volume (ITV). There was no expansion for microscopic disease. A 5-mm isotropic margin was added to form the planning target volume (PTV). The radiotherapy plan was calculated on the CT average image set and optimized using 7-10 beam angles. Intensity-modulated radiation therapy (IMRT) was used since 2009. The institutional policy was to deliver 48-52 Gy/4 fractions (fx) for peripheral NSCLC tumors (48 Gy if ≤ 3cm, 52 Gy if > 3cm) and 50 Gy/5fx for all central tumors (defined as tumors immediately adjacent to the esophagus, trachea, main stem bronchi, great vessels, and/or heart), regardless of size or histology. Plans were optimized to aim for ≥ 99% of the ITV to receive the prescription dose (ITV V100 ≥ 99%), and ≥ 99% of the PTV to receive 95% of the prescription dose (PTV V95 ≥ 99%). Radiotherapy plans were corrected for tissue inhomogeneity using the collapsed cone convolution algorithm.

Treatment was delivered using the Elekta Synergy units (Elekta AB, Stockholm, Sweden) equipped with the Elekta Synergy Beam Modulator (high resolution 4 mm multi-leaf collimator), a kilovoltage cone-beam CT (CBCT) image-guidance system and the Hexapod robotic couch permitting 6 degrees of freedom patient positioning.

**References**

1. Cook, G.J. *et al.* Non-Small Cell Lung Cancer Treated with Erlotinib: Heterogeneity of _18_F-FDG Uptake at PET-Association with Treatment Response and Prognosis. *Radiology.* **276,** 883-893 (2015).
2. Thibault, I. *et al.* Predictive factors for local control in primary and metastatic lung tumours after four to five fraction stereotactic ablative body radiotherapy: a single institution's comprehensive experience. *Clin Oncol (R Coll Radiol).* **26,** 713-719 (2014).
